# Address

**Published:** 1889-11

**Authors:** E. G. Betty


### Address.

BY E. G. BETTY,

President Seventh Ohio District Dental Society, held Sept. 17th, 1889, in Cincinnati, Ohio.

GENTLEMEN AND FELLOW MEMRERS:—

As president of the Seventh District Ohio Dental Society I feel it my duty to present a few words upon the work so far accomplished by this district society, for the benefit of those who are present for the first time at its meetings. A year ago in this hall a committee was appointed by our State Society to divide the State into suitable districts, and organize in each of them a society auxiliary to the State body. This work has been pushed until, I believe, nearly, if not all, the districts have completed their organization, each adopting a constitution and by-laws promulgated by the State Committee. This method bands together the profession of the entire State, and gives it the ability to act as a unit upon any question that may arise needing the combined action of all; this is a power you can readily appreciate. The work of each district, as I understand it, is to be reported to the State Society by some one conversant with it, the president or secretary, I presume. Anything of especial value done in any one of the districts, may with perfect propriety be brought before the parent body, or exchanged with some other district; in this way things of importance will gain a much wider notice than if allowed to go unheeded in the hurry and bustle of the State organization.

This district will, at this meeting, do a creditable share of work in the way of papers, Drs. J. Taft, C. M. Wright, L. E. Custer, J. R. Callahan, and others having prepared subjects for our consideration.

As regards the work we are to do, I would suggest that each and every one present in writing some thoughts on any of the multitudinous subjects of daily practice, new "tricks," instruments, appliances, and methods, together with an exhibition of the same. We hope to establish clinics wherein our operators can demonstrate their methods of operating.

Another point, and I have done. As Secretary of the Ohio State Dental Examining Board, I have received numerous applications from other boards and State societies for a roster of the State. This we do not possess, and it is my desire that this society shall take the initial step in that direction. A committee can be appointed in each county of the district whose duty it will be to make a complete list of all persons in his county engaged in the practice of dentistry, giving the name, status, *i. e.*, whether graduated or licensed, or either, his location, and if possible, the year in which he commenced practice. This list the committeemen can send to the president of his district, and through him, report to the State Society for publication. I doubt not but that this great State of Ohio will show a goodly number of practicing dentists, and I trust that the district societies will accomplish what the State body has failed to do, viz: secure the enactment of an adequate dental law.

Let us then, gentlemen, put the best foot forward and set a worthy example to the other districts, and make the old State Society think that she has unwittingly come to reside in this corner of the State.

